# Thwarted credibility work and double binds in medically unexplained pain in Japan: a qualitative study

**DOI:** 10.1016/j.pecinn.2026.100503

**Published:** 2026-09-09

**Authors:** Tsuyoshi Okuhara, Hiroko Okada, Rie Yokota, Yumi Kagawa

**Affiliations:** a Department of Health Communication, School of Public Health, Graduate School of Medicine, The University of Tokyo, Tokyo, Japan; b Department of Medical Communication, School of Pharmacy and Pharmaceutical Sciences, Hoshi University, Japan; c Psychology and Behavioral Sciences Division, Department of Human Sciences, Center for Liberal Arts and Sciences, Iwate Medical University, Japan

**Keywords:** Credibility work, Epistemic injustice, Medical invalidation, Chronic pain, Patient-centered care, Shared decision making, Health communication

## Abstract

**Objective:**

This study examined how people living in Japan with medically unexplained pain (MUEP) perform credibility work across clinics and everyday life, where and why credibility work falters, and the interactional double binds organizing these failures.

**Methods:**

We qualitatively analyzed first-person illness narratives from 16 adults with MUEP in the DIPEx-Japan Chronic Pain Archive, including cases of fibromyalgia, complex regional pain syndrome, chronic pain disorder, and undiagnosed persistent pain. An inductive reflexive thematic analysis was conducted using credibility as a guiding sensitizing concept.

**Results:**

Credibility work appeared cyclical and attritional rather than a direct route to belief. Information-seeking and efforts to translate invisible pain met test-centric norms and role expectations. During treatment negotiations, scarce options and firm professional boundaries narrowed shared decisions, leading patients to titrate what to say and when. Defensive practices (appearance normalization and economizing talk) thinned the information reaching clinicians, ending in expectation deflation and withdrawal. Recurring double binds organized this drift as follows: search suppression versus delegation, clinical translation versus test supremacism, preference expression versus resource constraints, and appearance normalization versus preventive silencing.

**Conclusion:**

In Japanese MUEP care, diagnostic uncertainty and power imbalance shift credibility work from trust-building to damage limitation. Clear communication, recognition of non-test evidence, and concrete ‘next-step’ planning can help sustain credibility.

**Innovation:**

We extend credibility work by identifying four MUEP-specific double binds and showing how scarcity and hierarchy invert credibility.

## Highlights

• Applies credibility work into medically unexplained Japanese pain narratives.
• Identifies four double binds structuring efforts to be believed.
• Credibility work can be defensive under scarce and asymmetric condition.
• Links micro-interactions to institutional constraints via epistemic injustice.
• Offers guidance for credibility-supportive communication and service design.

## 1 Introduction

Chronic pain, a complex sensory and emotional experience affecting approximately 15%–43% of adults worldwide [1], [2], is often accompanied by depression, anxiety, insomnia, and social isolation [3]. Beyond physical suffering, it disrupts identity, relationships, and the ability to communicate one's suffering as credible and worthy of clinical attention [4], [5]. Patients frequently encounter barriers to communicating their pain credibly, that is, to having their accounts accepted as believable and clinically relevant by providers, leaving them feeling misunderstood or hesitant to seek help [5], [6], [7], [8], [9], [10], [11]. Cheston [8] highlighted how medically unexplained symptoms invite skepticism from clinicians, evoking feelings of shame and undermining patients' self-understanding. These experiences eroded their sense of legitimacy as “credible narrators” in clinical settings [8].

Approximately 10% of chronic pain patients experience medically unexplained pain (MUEP), often marked by acute communication difficulties [12]. MUEP includes conditions such as fibromyalgia, complex regional pain syndrome (CRPS), chronic fatigue syndrome, and somatoform pain disorder, characterized by diagnostic uncertainty and a lack of clear biological markers. Patients with MUEP encounter disbelief from clinicians and must repeatedly justify their pain [4], [13]. Under such circumstances, pain becomes more than a symptom, undermining the individual as a communicative subject.

People with MUEP mobilize diverse interactional practices in clinical settings to secure legitimacy and maintain access to treatment and reasonable accommodation [5], [6], [8], [9]. These practices include visualizing symptoms, presenting material and pragmatic forms of evidence, translating experiences through metaphors and everyday enactments, managing emotions and appearances, and articulating and negotiating treatment preferences. Previous studies have collectively theorized these as credibility work [14], [15]. Credibility work is defined as the activities and efforts patients engage in to be believed in, understood, and taken seriously [14]. Credibility work denotes efforts that, in contexts where institutional and cultural biases readily cast patients' accounts as suspicious, aim to enhance the perceived credibility of those accounts and enable participation in clinical decision-making.

In clinical practice, several factors can undermine credibility work: diagnostic norms that equate negative test results with absence of pathology; limited resources for rare or novel symptoms; and communication norms defining how a “proper patient” should behave [4], [7], [10]. These factors often intersect with discussions of testimonial injustice [16], [17], [18], and medical invalidation/gaslighting [19], [20], [21], [22], both of which weaken the standing of experiential knowledge. Previous studies further suggest that in the course of undertaking credibility work, patients may be simultaneously exposed to conflicting expectations, that is, double binding [15]. However, context-specific descriptions remain limited regarding how such constraints arise from interactional and institutional conditions, and how they shape patients' practices: framing their narratives, efforts to make symptoms visible, negotiations around treatment, and the regulation of affect and appearance.

Compared to Western populations, Japanese people are generally less likely to make pain overt via facial displays, verbal complaints, or help-seeking, and there is a cultural tendency to valorize restraint in pain expression [23]. They also commonly endorse beliefs emphasizing not burdening others or deferring care to clinicians [24]. Studies of credibility work have been conducted predominantly in Western healthcare contexts, where norms around patient assertiveness, shared decision-making, and help-seeking differ substantially from those in Japan. How these distinctively Japanese cultural scripts (valorizing pain restraint, deference to clinical authority, and not burdening others) intersect with credibility work in MUEP, and thereby shape how it is practiced and where it breaks down, remains largely undescribed. Examining this gap matters because cultural norms do not merely provide background context; they may actively amplify or reconfigure the double binds that patients face in clinical encounters.

This study aimed to examine Japanese MUEP narratives through the lens of credibility work, focusing on how it is configured and mobilized in clinical and everyday interactions. We identified the clinical, institutional, and cultural conditions shaping these processes and discussed their implications for patient–clinician decision-making and access to care, with attention to interactional tensions and contradictions.

Thus, the guiding research questions for this study were:

RQ1 What forms of credibility work do patients with MUEP in Japan mobilize in clinical encounters and everyday life?

RQ2 What clinical, institutional, cultural, and interactional conditions cause credibility work to falter?

RQ3 What interactional double binds organize the breakdown of credibility work in the context of Japanese MUEP care?

## 2 Methods

### 2.1 Theoretical framework

Our analysis adopts a hermeneutic approach underpinned by critical realism [25], [26], consistent with the ontological flexibility of reflexive thematic analysis [27]. From this perspective, pain exists as a real phenomenon, even when it eludes full capture by biomedical indicators. It is only partially observable and is always interpreted within cultural and relational settings. In such settings, the credibility ascribed to accounts of pain depends on the context in which people tell their stories and how clinicians read them. Analytic sensitivity draws on the credibility work literature [14], [15], with particular attention to the repertoire of credibility work strategies, moments when those strategies break down, and the double binds that accompany credibility work.

### 2.2 Participants and procedures

Participants were selected from the DIPEx-Japan Chronic Pain Archive (*n* = 42) [28], a systematically curated repository of first-person illness narratives developed initially in the UK and now maintained internationally for research, teaching, and public engagement.

We excluded individuals with clearly established biomedical diagnoses such as rheumatoid arthritis, lumbar disc herniation, and endometriosis (*n* = 26). The final analytic set comprised 16 adults living with medically unexplained or etiologically opaque pain (MUEP): 69% women; age 22–61 years (M = 43.8, SD = 8.8); pain duration 2–42 years (M = 12.2, SD = 10.3). Reported labels included fibromyalgia (*n* = 7), complex regional pain syndrome (CRPS; *n* = 4), chronic intractable pain (*n* = 1), chronic fatigue syndrome (*n* = 1), and chronic pain disorder (*n* = 1); two participants described persistent symptoms without any diagnosis (Table 1).

**Table 1:** Participant characteristics (*N* = 16).

Participants ID	Age	Gender	Pain duration	Diagnosis
#1	45	Male	5	Fibromyalgia
#2	40	Female	20	Fibromyalgia
#3	43	Male	23	Fibromyalgia
#4	44	Female	5	Fibromyalgia
#5	49	Female	42	Fibromyalgia
#6	55	Female	14	Fibromyalgia
#7	57	Female	4	Fibromyalgia
#8	22	Female	2	CRPS
#9	61	Male	14	CRPS
#10	45	Female	3	CRPS
#11	47	Female	9	CRPS
#12	36	Female	6	Chronic intractable pain
#13	42	Female	11	Chronic fatigue syndrome
#14	41	Female	7	Chronic pain disorder
#15	30	Male	15	Undiagnosed
#16	45	Female	8	Undiagnosed

CRPS: complex regional pain syndrome.

Interviews were conducted by trained DIPEx-Japan researchers between June 2015 and June 2017 using a semi-structured guide. The present authors accessed the archived transcripts for secondary qualitative analysis; we did not conduct the original interviews. Open-ended prompts invited narratives about the onset and course, care seeking and treatment, interactions with clinicians and others, everyday impacts, and sense-making. The total interview time was 36 h 56 min, and individual interviews ranged from 54 min 14 s to 219 min 28 s (M = 123 min 06 s, SD = 52 min 17 s), producing verbatim transcripts of 8–48 A4 pages (M = 26.1, SD = 10.7). All participants provided informed consent at the time of the original interviews. Ethical approval for secondary analysis was obtained from the DIPEx Japan Ethics Committee (ID: DS24-02).

### 2.3 Analysis

We used a reflexive thematic analysis (RTA) [27], which foregrounds both interpretive work and theoretical engagement. Following Braun and Clarke's six phases [27], we moved iteratively through familiarization, open coding, theme construction, review, definition, and analytic narration. The lead author conducted primary coding, privileging depth and reflexivity over intercoder agreement, consistent with RTA. An evolving codebook was maintained through memos and iterative revisions using word processing and spreadsheet tools. Semantic codes captured explicit statements (e.g., “I stopped saying ‘it hurts’”), while latent codes traced underlying sociocultural dynamics (e.g., preventive silencing via outward normalization). The codes were then clustered into candidate themes and refined through constant comparisons within and across cases.

Team discussions were crucial to test and elaborate interpretations. Credibility was supported by reflexive memos, documentation of analytic decisions and shifts, peer debriefing, an audit trail, and a thorough description of the context and excerpts.

### 2.4 Positionality and reflexivity

Reflexivity was integral to the study design and analysis. The first author, trained in health communication and narrative inquiry, approached the material with both professional and experiential familiarity with chronic pain through long-term proximity to a spouse living with rheumatoid arthritis. This insider/outsider positioning shaped the analysis in concrete ways. The insider perspective, grounded in lived proximity to chronic pain, enabled the first author to imaginatively inhabit participants' suffering and isolation, fostering an empathic attunement to experiential dimensions that may be less perceptible from a clinician's standpoint. This sensitivity sharpened attention to moments of withdrawal, silence, and expectation deflation as meaningful analytic signals rather than absences of data. Simultaneously, the outsider perspective, as a trained researcher, provided the critical distance necessary to situate these empathic readings within theoretical and structural frameworks, translating felt resonance into analytically grounded interpretations. To ensure this dual positioning enriched rather than distorted the analysis, the team engaged in periodic bracketing discussions in which the first author made explicit how personal resonance was influencing emerging codes, allowing co-authors to interrogate and reframe interpretations toward latent, structural levels. Reflexive memos documented instances where initial codes risked over-identifying with participants' distress; peer debriefing sessions prompted re-examination of these codes, ultimately shaping the conceptualization of double binds as institutional rather than purely interpersonal phenomena.

## 3 Results

The following five themes address our three research questions, but not in a one-to-one mapping. Themes 3.1 through 3.4 are each organized around one domain of credibility work, and each follows the same internal sequence. Each theme first describes the forms of credibility work that patients mobilized in that domain (RQ1). It then describes the clinical, institutional, cultural, and interactional conditions under which those forms faltered (RQ2). It then identifies the double bind that this collision produced (RQ3). All three research questions are therefore addressed within each of these four themes, and RQ3 is not confined to Theme 3.5. We named Themes 3.1 through 3.4 after the double binds because our analysis identified the bind, rather than the practice or the condition alone, as what organizes the relation between the two. Each title accordingly pairs a domain of credibility work (information seeking, making pain visible, treatment negotiation, and appearance and talk) with the bind that this domain produced. Theme 3.5 does not introduce a fifth bind. It traces the cumulative consequence of the four, showing how repeated exposure across encounters settles into expectation deflation and withdrawal.

Together, the themes trace a single cyclical dynamic unfolding across all five, in which each breakdown feeds into the next. Patients' initial moves (information seeking and the translation of invisible pain) collided with test-centric norms (the institutional tendency to equate diagnostic validity exclusively with objective test results) and role expectations. In negotiation, scarce options and firm professional boundaries further narrowed the narrative space (the interactional room available for patients to articulate their experiences and preferences). To preserve relationships and access, patients fine-tuned when and how to speak and adopted defensive practices (appearance normalization and economizing talk), which thinned the information reaching clinicians and fueled a negative feedback loop toward expectation deflation and withdrawal. Four recurrent double binds organized this drift: (1) “*don't look it up/look it up yourself*,” (2) “*visibility demanded/discounted by negative tests*,” (3) “*advocate* = *noncompliant/defer* = *powerless*,” and (4) “*look normal* = *not a real patient/display pain* = *difficult*.”

### 3.1 Double bind of information seeking: “*don't look it up/look it up yourself*”

This theme shows a double bind: patients' information seeking (doing research), undertaken as credibility work to bolster the credibility of their accounts, [15] is discouraged by clinical discourse, yet, when care stalls, recast as the patient's responsibility (“*look it up yourself*”). Assuming “*lay searching is harmful*” (clinicians' epistemic superiority and a monopoly on objective information), clinicians view such searches with suspicion; conversely, in resource-constrained moments (“*there's nothing more medicine can do*”), the same activity is urged. One patient reported a physician withholding the diagnosis, citing concern about the patient's internet searching. The following account illustrates how a physician explicitly framed the withholding of a diagnosis as protection from the patient's own information-seeking:

*“By assigning a diagnosis, you might start looking things up a lot on your own. The internet is full of information—some trustworthy and some not—so they said they didn't want me exposed to that. They told me they wanted to avoid a situation where, after I looked things up myself, I'd be told something that would create new assumptions, my symptoms would worsen, or new symptoms would appear.”*

*(Participant #8)*

This account reveals how clinical authority was used to preemptively restrict the patient's epistemic access, framing the patient's own curiosity as a clinical risk.

Another patient reported that, when the physician informed her that treatment was being discontinued, the physician added, “Figure it out by researching on your own.”

*“We ran several tests, and I was even prescribed Kampo medicine, but nothing had any effect. Eventually, the doctor said, ‘Let's stop now.’ I wondered what exactly we were stopping, and it turned out he meant stopping further tests and treatment. In the end, he told me, ‘Search on your own and find symptomatic treatments that suit you.’ Hearing that from my doctor made me think I had nowhere left to go, and I became extremely pessimistic.”*

*(Participant #16)*

Here, the physician's final instruction, “Search on your own,” inverts the earlier suppression of searching, making the patient responsible for finding solutions the clinic could not provide.

As above, clinicians both suppressed patients' searches (“I didn't want you exposed to information”) and later delegated them (“*look it up yourself*”). Thus, information seeking faces a dilemma: search and be suspected, do not search, and have no next step. This dynamic fostered uncertainty and self-doubt, progressively nudging patients toward withdrawal, silence, and stalling of care. For people with MUEP, credibility work can therefore precipitate termination rather than strengthening of the patient–clinician relationship.

### 3.2 Double bind of making pain visible: “*visibility demanded/discounted by negative tests*”

This theme illustrates how patients mobilize credibility work by “*clinically translating*” invisible pain into metaphors, demonstrations of everyday functioning, family testimonies, and images to help render their accounts credible within medical interpretive frames. At the same time, entrenched norms equating negative tests with no disease and routine denial of rare presentations place these visibility efforts in conflict with the diagnostic authority. This constitutes a double bind in which simultaneous demands for visibility and dismissal based on negative tests operate together, systematically discounting the “*evidence languages*” that patients bring.

The following account illustrates the psychological reframing of physical pain that patients encountered when seeking clinical recognition:

*“What hurt most was that the doctors treated me like, ‘Are you really in pain?’ And when I was taking things like adjuvant analgesics—tranquilizers—they looked at me everywhere as if, ‘Isn't the real problem psychological?’ That was very hard.”*

*(Participant #12)*

This account shows how patients' credibility work, seeking to have pain recognized as physically real, was undermined by a psychologizing clinical response that reframed somatic suffering as emotional. A different form of dismissal appears in the next account, where the rebuff drew not on psychology but on the absence of precedent.

*“I told the doctor that the pain had gotten to the point where I couldn't breathe. But when it came to proving that—this has happened with other illnesses too—the doctor said, ‘There are no such cases.’ I've been told that many times.”*

*(Participant #5)*

The repeated dismissal “*there are no such cases*” illustrates how clinical epistemology, privileging established precedent over individual experience, renders patients' pain accounts structurally incredible.

This kind of self-clinicalization, where patients learn pharmacology, track symptoms, and undertake planned self-adjustments, is a highly skilled form of credibility work. However, it is often perceived as an overstepping of authority or non-adherence, which widens rifts in the patient–clinician relationship.

*“For about two years, I was on the maximum dose of antidepressants because the doctor treated it as psychological. Even when my body began moving on its own—a serious sign—I was largely left as is. I wanted to taper in consultation, but the doctor kept wanting to try new drugs, so prescriptions continued. When a drug felt wrong, I checked pharmacology references such as peak levels and decline, tracked my body for a week, then tapered myself in a planned way—essentially an experiment—and felt better off the medication. I told the doctor I couldn't keep taking it and that this worked better. It wasn't really a consultation; I had to take the lead in stopping, which created a rift between us.”*

*(Participant #2)*

This account demonstrates how self-clinicalization, a sophisticated form of credibility work, was reinterpreted as non-adherence, transforming the patient's expertise into evidence of problematic behavior.

Across these accounts, patients repeatedly mobilized diverse forms of evidence, including symptom records, functional demonstrations, and family testimonies, to make invisible pain legible within clinical frames. Yet these efforts were systematically met with dismissal grounded in negative test results or psychologizing responses, rendering credibility work an accumulating and unreciprocated labor [15].

### 3.3 Double bind of treatment negotiation: “*advocate* = *noncompliant/defer* = *powerless*”

Patient narratives show that when people justify side effect concerns or request stronger analgesia, clinicians invoke sequencing, policy, and professional boundaries, sometimes with rebukes. When patients in severe, uncertain pain “*leave it to the doctor*,” they are told that options within approved therapies are scarce. As credibility work, patients keep recalibrating how much to speak and when to defer, yet these constraints further narrow negotiations, forcing fine-tuning of framing, reasons, and affect. This yields a double bind: “*advocate* = *noncompliant; defer* = *powerless.*” This shrinks the space for shared decision-making.

The following account illustrates how expressing a treatment preference, a core form of credibility work, was met with a sanction that closed off further negotiation:

*“I told the physician I would prefer to avoid that medication—I'd been experiencing nausea and didn't want to take it. In the end, though, biomedicine meant pharmacotherapy, and I was told I should take it. When I then said I really couldn't, I was told, ‘Don't cross this threshold again’ [i.e., don't come back]. I understood that, within a relationship of trust, I was supposed to follow the doctor's instructions, but I was still scolded like that.”*

*(Participant #16)*

Here, the patient's refusal of a medication, grounded in experiential knowledge of side effects, was treated as a violation of the therapeutic relationship rather than as legitimate clinical input.

*“After receiving the diagnosis I was so anxious that I wanted to try this, try that—I kept pressing, thinking there must be some other treatments. I even got angry when they wouldn't provide the treatment I wanted. When they said no, I said, ‘No, that won't do,’ ‘Absolutely not.’ Even crying didn't change anything.”*

*(Participant #11)*

This account illustrates how emotional intensity, a form of nonverbal credibility work, failed to shift clinical decision-making, reinforcing the patient's sense of powerlessness.

*“At that time in Japan, the only approved drug was pregabalin (Lyrica), and I was already on the maximum dose, plus steroids. But the pain didn't ease. I asked for stronger pain relief, but the doctor told me clearly, ‘These are the only medications approved in Japan, and there's nothing else we can do.’ … The pain was beyond my control, so I had no choice but to entrust myself to the physician's treatment plan.”*

*(Participant #4)*

Across these accounts, patients continually recalibrated how much to say and when to defer, yet both strategies narrowed the space available for shared decision-making. Advocacy was read as noncompliance while deference exposed a paucity of options, progressively eroding patients' bargaining power [15].

Participant #16's own account is telling: she understood that, within a trusting relationship, she was supposed to follow the doctor's instructions. This shows how deference to clinical authority is internalized as a relational obligation rather than experienced only as a clinical constraint. Such internalized deference narrows the already limited space for preference expression and sharpens the double bind of “*advocate* = *noncompliant/defer* = *powerless*.”

### 3.4 Double bind of appearance and talk: “*look normal* = *not a real patient/display pain* = *difficult*”

What emerges are practices such as describing only observable phenomena instead of saying “*it hurts*,” maintaining a normal demeanor in workplaces and clinics, and “*reading the room*” to withhold complaints. Aware of the look-fine/felt severe-pain gap, the participants preemptively curtailed talk to avoid poor legibility or social exclusion. As credibility work, these interpersonal adjustments minimize friction and preserve access. The side effects include thinner information reaching clinicians, more challenging pathways to understanding and support, and intensified isolation with a sense that “*there's no point in saying it*.” A double bind also operates: looking put-together reads as “*not a real patient*,” while foregrounding pain attracts a “*difficult*” label, prompting further preventive shrinkage of talk.

The following account captures the core tension of this double bind, the gap between outward appearance and internal experience that renders pain invisible and self-expression futile:

*“The gap with appearance is a huge barrier. Given how much I say my body hurts, I don't show ‘painful’ gestures and I don't look like I'm in pain. That gap is really hard. Even if I say I live with this much pain, I'm not believed, and it's mentally exhausting. My appearance is normal, but my body hurts a lot. I work and move as usual, so I end up unable to say it even when I want to. … Even if I say ‘it hurts’ when the pain is severe, pain isn't visible, so it doesn't get across. Even if I grimace and say ‘it hurts,’ people don't understand.”*

*(Participant #15)*

This account illustrates how the gap between appearance and experience becomes self-reinforcing: the very success of appearance normalization (looking functional) undermines the patient's credibility as a person in pain.

The following two accounts from the same participant illustrate how economizing talk operates in both social and clinical contexts: first, as a withdrawal from pain disclosure when it is met with indifference; and second, as a substitution of functional language for pain language to avoid burdening others.

*“With chronic pain, in the end nobody worries about you, no matter how much you say it hurts. People think, ‘Well, there's nothing we can do, and it's not life-threatening.’ So there's no point saying it—no point complaining.”*

*(Participant #11)*

*“I don't tell people at work at all. I just say things like, ‘Sorry, I can't reach up right now.’ I don't say it hurts. I've said, ‘My toes don't work well, so reaching up or going down stairs is hard for me,’ but I've never said it hurts.”*

*(Participant #11)*

Together, these accounts show how the credibility work of economizing talk functions as a learned adaptation: the patient anticipates disbelief and social inconvenience, and pre-emptively contracts her disclosure to avoid both.

*“If I just endure it, other people won't notice, so maybe that's fine.”*

*(Participant #8)*

This brief account encapsulates the logic of preventive silencing: endurance becomes rational precisely because visibility offers no benefit.

These accounts depict a paradox in which interpersonal accommodations, maintaining a normal outward appearance and economizing one's talk, end up fostering difficulty in being believed in and reinforcing self-silencing. In this context, “*managing appearance*” as credibility work [15] functions not as a strategy to strengthen trust and understanding but as a defensive practice to avoid relational rupture or penalties (exclusion, reprimand, being labeled “*difficult*”). As a side effect, informational gaps widen in clinical encounters and social isolation deepens.

These accounts make the cultural patterning concrete. Participant #8 chose to endure so that others would not notice, and Participant #11 avoided saying “it hurts” even at work. In both, appearance normalization and economizing talk enact the cultural script of gaman (endurance), in which suppressing visible pain is treated as the proper and considerate response. Read this way, these practices are not merely individual coping strategies but culturally structured ones that systematically thin the information reaching clinicians and others.

### 3.5 From expectation deflation to withdrawal: the cumulative toll of double binds

This theme traces how repeated denial, resource constraints, labeling, and gatekeeping push patients toward an expectation-deflation stance, choosing not to seek understanding and presuming that they cannot be understood, and ultimately toward withdrawal-based survival (interrupting clinic attendance, contracting one's narrative, and social isolation). From the standpoint of credibility work, practices originally intended to enhance relational trust and the credibility of one's account are worn down by double binds, such as “*don't look it up/look it up yourself*” and “*if you advocate you're noncompliant/if you defer you're powerless*,” so that self-restraint and silence become structured as rational choices.

*“It's easier not to have excessive expectations. People often talk about ‘mutual understanding,’ but, in the end, we're human precisely because we don't fully understand one another—and that distance is how we keep going. Of course I want others to understand me, to really get it, but holding on to that too much just makes it harder. Lately I think it's better to proceed on the assumption that people can't fully understand one another.”*

*(Participant #2)*

For others, lowering expectations hardened into relinquishing the wish to be understood at all.

*“Wanting others to ‘please understand’ is itself mistaken. I don't even understand it myself. My husband, who helps with my daily life, doesn't understand why either. I don't know why this pain happens or why it is so unbearable, so I no longer ask others to understand— I don't even want them to.”*

*(Participant #6)*

This relinquishment, in turn, settled into a practiced silence.

*“Now I just endure and stay quiet. Talking it over doesn't really go anywhere—we stay on parallel lines—so all I can do is put up with it. After repeating that again and again, I've pretty much stopped telling other people about myself.”*

*(Participant #1)*

These accounts present a clear sequence of withdrawing from medicine and social life. As a rational strategy to avert relational rupture and preserve basic survivability, patients are steered toward contracting their talk, interrupting care seeking, and accepting emotional isolation. In this context, the credibility work of “*tempering expectations*” [15] operates not to secure trust, but as a technique of strategic retreat to minimize attrition, thereby inadvertently reproducing informational gaps in the clinic and discontinuities of care.

## 4 Discussion and conclusion

### 4.1 Discussion

Our findings reveal that the credibility work among patients with MUEP frequently collapses into double binds. Although intended to make their accounts believable, these efforts often backfire amid diagnostic uncertainty, privileging of negative tests, professional boundary maintenance, and resource constraints. This study is consistent with Thompson et al.'s characterization of credibility as a continual labor of constructing relationships and explanations [14], and with work on Mono−/Multiracial women of color in the United States with autoimmune diseases [15]. Both bodies of work document contexts in which credibility work is institutionally disenfranchised; the present study makes visible, in concrete terms, how this faltering unfolds under MUEP-specific constraints and how patients' narratives are progressively thinned in the process.

This study supports Gunning's observation that patients face a paradox: when they speak, their accounts are reinterpreted as emotional excess or psychogenesis, yet when they remain silent, they are read as an absence of symptoms or non-cooperation [15]. We identified four interrelated double binds recurring across settings: *don't look it up/look it up yourself*; *visibility demanded/discounted by negative tests*; *advocate* = *noncompliant/defer = powerless*; and *look normal = not a real patient/display pain = difficult*. These binds imposed a continual demand on patients to calibrate when and how to speak. Over time, this cumulative pressure rationalized expectation deflation and withdrawal from care.

At first glance, the double binds identified in this study may appear distinct from the dialectical tensions theorized in early relational dialectics theory (RDT 1.0) [29], which centered on opposing relational desires, such as autonomy versus connection, that partners recognize and negotiate dialogically. Yet the theory's later reformulation (RDT 2.0), advanced by Baxter from 2011 onward, offers a closer point of contact [30], [31]. RDT 2.0 relocates the analytic focus from individuals to the interplay of competing discourses, including the “distal already-spoken” at which everyday talk meets broader cultural and institutional systems of meaning, such as the contending discourses of speaking up and remaining silent. Read through this lens, the Japanese cultural scripts traced here (valorizing pain restraint, deference to clinical authority, and not burdening others), together with test-centric institutional norms, function as precisely such competing discourses that patients must navigate in clinical talk; the double binds we document can thus be situated within, rather than apart from, the multiple interpersonal and cultural discourses that health interactions require patients to manage. What the present findings add is twofold. First, these binds are tightly coupled with credibility work: the very practices patients mobilize to make their accounts believable activate the binds, so that credibility work simultaneously constitutes and is undermined by the double-binding structure. Second, the power asymmetry inherent in clinical encounters forecloses the meta-communicative reframing that RDT treats as a resource for managing discursive struggle [32], leaving patients little viable position from which to challenge the bind itself. In this sense, the binds described here are less open to dialogic transformation than the discursive tensions RDT typically examines, marking a clinically specific extension of the theory rather than a departure from it [33].

Reinterpreting credibility work through Fricker's framework of epistemic injustice highlights its role as a counter technique to testimonial injustice [13], [16], [17], [34]. Patients mobilize diverse visibility practices, such as self-management records, indicators of everyday functional limits, nonverbal displays of distress, and timeline accounts of symptom trajectories, to repair credibility deficits in their testimony. However, these counter techniques are frequently neutralized by the double binds described above and can even reproduce new credibility deficits. Moreover, the requirement to continue translating pain into biomedical terms within test-centered validity regimes points to a hermeneutical injustice in which skewed interpretive resources prevent patients' experiences from being properly located [4], [10], [35]. Thus, the breakdown of credibility in MUEP care demonstrates how institutional norms invert these counter techniques, simultaneously sustaining testimonial injustice and deepening hermeneutical injustice.

From the perspective of medical invalidation, the breakdown of credibility work and the attendant double binds can be understood as the cumulative effect of invalidating practices: symptom minimization, psychologization, overreliance on negative test results, and refusal of treatment [19], [20], [21]. Even when patients refine their accounts and multiply their “*evidence languages*,” repeated clinical replies such as “*there are no such cases*,” “*it's in your head*,” or “*we don't treat that here*” prevent credibility work from gaining traction and instead rationalize expectation deflation, the sense that speaking up is futile [15]. In this respect, the observed dynamics closely resemble medical gaslighting [22]; they systematically erode confidence in one's own perceptions, shift causal attributions to the patient's psyche or character, and ultimately entrench self-doubt and silence.

Taken together, this study re-articulates the boundary conditions for credibility in the context of MUEP. Credibility work is not merely an individual endeavor, but a fragile practice that readily backfires at the intersection of institutional norms and interactional orders. In addition to the generic double bind noted by Gunning [15], the MUEP-specific double binds we identified tended to transform credibility work from a counter to testimonial injustice into a retreat technique aimed at damage limitations. Accordingly, the breakdown of credibility work and these double binds should be situated within Fricker's framework as the co-occurrence of testimonial and hermeneutical injustice [16], and within clinical sociology as recurrent patterns of medical invalidation [19], [20], [21] and gaslighting [22].

The phenomena of appearance normalization, economizing talk, and expectation deflation identified in this study are not merely individual coping strategies but culturally structured responses shaped by distinctively Japanese norms and institutional arrangements [23]. Such suppression norms foster preventive silencing internalized as self-discipline (“*not saying or showing pain*”), thereby pushing credibility work toward defensive practices that dampen and invisibilize pain expression. Moreover, qualitative research on Japanese patients with knee osteoarthritis shows that pain is “inevitable with aging,” alongside the strong values of not burdening others and deference to clinicians [24].

These Japanese cultural norms further complicate credibility work in clinical encounters and can intensify a cultural double bind: the more patients minimize their complaints, the more they are read as “*not patient-like,*” whereas the more they amplify them, the more they are judged “*difficult.*” Supporting credibility work in Japanese clinical practice therefore requires cultural adaptation beyond generic communication techniques. This includes explicitly legitimizing concrete talk about pain (e.g., “Please describe your pain in detail, as this is appropriate and clinically useful”) and instituting procedures that reduce reticence (e.g., pre-visit questionnaires or structured invitations such as “To start, tell me just one pressing concern”).

Beyond pain behavior norms, the Japanese clinical context is characterized by pronounced power asymmetry between clinician and patient, with shared decision-making remaining limited in practice compared to many Western healthcare settings [36], [37]. Deference to clinical authority is culturally embedded, such that patients who advocate for their preferences risk violating relational norms as much as clinical ones. In this context, the double bind of “*advocate* = *noncompliant/defer* = *powerless*” is not simply an interactional phenomenon but a structurally and culturally enforced constraint. Furthermore, the cultural norm of endurance (gaman) intersects with credibility work in a distinctive way: when patients internalize pain suppression as self-discipline, appearance normalization ceases to be a strategic choice and becomes a habituated practice, systematically thinning the information available to clinicians while rendering patients less recognizable as legitimate sufferers.

The following recommendations are informed by the findings of this study. We recognize, however, that several of these recommendations presuppose conditions (patient assertiveness, clinician openness to dialogue, and institutional flexibility) that may be structurally and culturally constrained in the Japanese context. From a culture-centered perspective [38], the capacity of both clinicians and patients to enact these practices is shaped by the intersection of cultural norms, structural constraints, and agency. In Japan, the cultural valorization of authority deference and pain suppression, combined with structural conditions such as short consultation times and resource-constrained care, may substantially narrow the space within which transparent communication and joint evidence-building are feasible. We therefore frame these recommendations not as universal prescriptions but as context-sensitive starting points requiring cultural adaptation.

Practically, clinicians should establish an explicit communication component at the outset to preempt double-binding. This entails transparent discussion of the diagnosis, uncertainty, and treatment limits while explicitly welcoming patients' searching and experiential knowledge as clinical resources (e.g., “bring what you've found; we'll review it together”), aligning role expectations and shifting credibility work from relationship-maintenance micro-adjustments to joint evidence-building [14], [15].

Integrate the visualization of “*invisible pain*” into routine workflows: systematically elicit, re-articulate, and document non-test evidence languages (metaphors, concrete functional disruption, family observations, episodic flares) within the record. This counterbalances the test-centric validity norms and mitigates testimonial injustice [10], [39]. To forestall dismissive responses, clinicians should explicitly incorporate epistemic injustice and medical invalidation frameworks with ongoing case-based learning and reflective practice on language and attitudes that hinder access [40], [41], [42].

Organizationally, standardize a “*what comes next*” checklist when communicating plan limits (near-term pain goals, referrals/second opinions, self-management) and provide default pathways to second opinions and communication support. Such an infrastructure reduces breakdowns and double binds in the credibility work of people with MUEP, serving as a primary prevention against testimonial injustice and medical invalidation/gaslighting.

This study had some limitations. First, it was conducted in a single national setting (Japan), where norms around pain stigma, care access, and clinician–patient roles may differ from those elsewhere, limiting transferability. The sample comprised people who were willing and able to narrate their experiences; voices muted by distrust, trauma, communication hurdles, or other barriers were likely underrepresented. As a result, the corpus reflects accounts that can be voiced and may miss deeper structural mechanisms that suppress narration. In addition, the material is retrospective and thus vulnerable to recall bias, although our emphasis on subjective meaning-making rather than factual reconstruction helps limit the threat to analytic claims. Finally, because we drew on an existing narrative archive, we could not purpose-build a dataset to comprehensively capture the full range of credibility work. Even so, the data were sufficiently rich to delineate diverse credibility work practices among patients with MUEP and the double binds that accompany them.

### 4.2 Innovation

This study offers conceptual, methodological, and empirical innovations. Conceptually, it extends the notion of credibility work. Thompson et al. theorized credibility work as the ongoing labor of building relationships and explanations [14], and Werner and Malterud documented the effort of behaving as a credible patient among women with chronic pain [5]. Gunning et al. showed how that labor is institutionally disenfranchised for Mono-/Multiracial women of color with autoimmune disease in the United States [15]. In these accounts, credibility work remains a resource for securing trust, however costly it is to sustain. Our findings show that under MUEP conditions it can turn attritional and defensive rather than trust-building. Practices that succeed on their own terms, such as maintaining a composed appearance, economizing talk, and lowering expectations, thin the information reaching clinicians. Both studies also documented double binds accompanying credibility work. Thompson et al. described the binds that arose as women built and presented their case [14], and Gunning et al. identified two binds tied to ethnic-racial identity enactment [15]. Neither set was specific to conditions in which no diagnosis is available. Using credibility work as an explicit lens within inductive reflexive thematic analysis, we specify four double binds particular to MUEP (search suppression/delegation, clinical translation/test supremacism, preference expression/resource constraints, and appearance normalization/preventive silencing) and trace how they accumulate into expectation deflation and withdrawal.

Methodologically, we implemented a theory-informed inductive RTA [27] that remains grounded in participants' language while being anchored in credibility work [14], [15]. Credibility work has so far been developed through grounded theory [14] and phronetic iterative qualitative data analysis [15]. Our design complements these by enabling fine-grained mapping from utterances to meso-level mechanisms. More broadly, credibility work has been elaborated almost entirely in Western, and predominantly United States, care settings [14], [15]. Adapting it to Japanese MUEP narratives demonstrates its portability and also shows that it is not culturally neutral. Norms valorizing restraint in pain expression [23], beliefs about not burdening others and deferring to clinicians [24], and the limited uptake of shared decision-making in Japanese practice [36], [37] actively tighten the binds themselves. Under these conditions, appearance normalization shifts from a strategic choice to a habituated practice. Finally, we link micro-interaction to institutional constraints through epistemic injustice [16], [17], [18] and medical invalidation and gaslighting [19], [20], [21], [22]. We translate this account into actionable guidance for credibility-supportive communication and service design, including routine documentation of non-test evidence languages [10], [39] and case-based clinician education on invalidating language [40], [41], [42].

### 4.3 Conclusion

For patients in Japan with MUEP, credibility work across clinical and everyday contexts readily turns (amid diagnostic uncertainty, scarce treatment options, and asymmetric authority) into attritional and defensive labor. Four recurring double binds progressively thin efforts in information seeking, translating invisible symptoms, negotiating treatment, calibrating appearance/talk, and ultimately rationalizing expectation deflation and withdrawal. We reposition credibility work from an individual trust-building tactic to a fragile practice easily countered by institutional norms and interactional orders and offer a typology of MUEP-specific double binds. These dynamics function as circuits that sustain testimonial disadvantages, skew interpretive resources, and cause repetitive medical invalidation/gaslighting. Clinically, fostering credibility-supportive care requires embedding recognition and practical support in routine care, professional education, and service design.

## CRediT authorship contribution statement

**Tsuyoshi Okuhara:** Writing – review & editing, Writing – original draft, Methodology, Investigation, Funding acquisition, Formal analysis, Conceptualization. **Hiroko Okada:** Writing – review & editing, Investigation, Formal analysis. **Rie Yokota:** Writing – review & editing, Investigation, Formal analysis. **Yumi Kagawa:** Writing – review & editing, Methodology, Investigation.

## Ethics declaration

Written informed consent to take part in the study and to publish the article has been obtained from all participants or their legal representatives. The privacy rights of participants have been observed.

This study was performed in compliance with relevant laws, regulatory frameworks and guidelines where the research took place.

This study was approved by the The DIPEx Japan Ethics Committee.

(Approval No. DS24-02)

## Funding

This study was supported by the 10.13039/501100001691Japan Society for the Promotion of Science, KAKENHI (grant number: 25K13483).

## Declaration of competing interest

The authors declare that they have no known competing financial interests or personal relationships that could have appeared to influence the work reported in this paper.
